# 

**Published:** 1890-04-26

**Authors:** 


### NEW OFFICES IN THE STRAND.

The Hospital *is now published at* **140, Strand, W.C.** *It has been found desirable to establish* The Hospital *in this central and convenient locality, owing to the rapid extension of the business of this paper of late.*

### NOTICE TO CORRESPONDENTS.

All MS., Letters, Books for Review, and other matters intended for the Editor should be addressed The Editor, The Lodge, Porchester Square, London, W.

The Editor cannot undertake to return rejected M.S., even when accompanied by stamped directed envelope.

**Notice to Advertisers and Subscribers.**

All Advertisements, Orders for Copies of The Hospital, and Business Communications should be addressed to The Publisher, *not to the Editor*, The Hospital, 140, Strand, W.C.

**Bound Volumes of "The Hospital."**

Vol. VI., containing full page portraits of T.R.H. the Prince and Princess of Wales, and Miss Florence Nightingale, is obtainable at 4s., post free.

Orders will be received for Vol. VII., 4s. post free. Cases for binding may be had of the Publisher, at 1s. 6d. each.

### Scraps and Gleanings.

Twenty lads have been sent to Canada by the Children's Aid Society.

A fresh wing has been added to the St. Raphael's Hospital for Consumptives at Worthing.

Three ladies from Australia have recently entered as students in the Calcutta Medical College.

The Newspaper Press Fund dinner will be held on June 7th, Mr. Edward Lawson in the chair.

Mr. Frederick Keyt has been appointed Third Assistant Medica Officer to Kent County Asylum.

Mr. W. C. Deverean has been appointed Assistant House Surgeon to the Birmingham General Hospital.

Another medical novel is announced, "The Modern Malady" by Cyril Bennett who wrote "The Massage Case."

Dr. Knorr, the discoverer and patentee of Antipyrin. is said to have cleared £200,000 during the influenza epidemic.

A testimonial is to be given to Mr. Samuel Sims to mark the appreciation of his forty years' work in the temperance cause.

Donations amounting to £1,563, were received after the annual dinner in aid of the Reedham Asylum for Fatherless Children.

The Duke of Westminster has given £100 towards the Seaside Camp for London Working Boys, to be pitched again this summer at Deal.

Dr. Symes Thompson lectured on "Heredity, or Inherited Characteristics," before a numerous audience at the Gresham College last week.

The Duchess of Teck has become the patron of the King Edward Ragged Schools Youths' Institute and Christian Mission, Spitalfields.

The New Hospital for Women will open to out-patients after the bazaar, commencing on the 29th; the wards will be open to in-patients in June.

Count and Countess Maulmont have been arrested in Paris, for collecting money for charities, and then using the money for private purposes.

The Convocation of York is going to petition Parliament on the subject of burial reform and the enforcing of sanitary precautions in the interests of the living. Why not cremation at once?

The influenza has spread throughout India. The troops, especially those of the hill stations, are suffering severely. The epidemic is generally of a mild type, but some deaths are reported.

Mr. J. W. B. Pogson, M.B., has been presented by the Mayor of Durham with a handsome silver-mounted stick, on behalf of the members of the Durham branch of the St. John Ambulance Association.

A bazaar, under the patronage of the Princess of Wales, in aid of the New Hospital for Women, will be opened at the hospital, 144, Euston Road, on Tuesday, the 29th inst., and remain open until May 1st.

Mr. V. E. Walker, of Arno's Grove, Southgate, has generously presented to the Southgate Local Board fifteen acres of land, situated at New Southgate, for the purpose of laying out public recreation grounds.

The nona has appeared in Dalmatia. The Austrian Government has instituted an inquiry into the nature of this mysterious disease, and ordered that in every fatal case a post-mortem examination shall be made

The Doll Show at the bazaar in aid of the New Hospital for Women promises to be a great success. Fifteen hundred dolls have been sent in, and they are to be judged and the prizes awarded the day before the opening.

On Tuesday, May 6th, at St. George's Hall, a dramatic entertainment will be given in aid of the Establishment for Invalid Gentlemen, 90, Harley Street. Tickets can be had from the lady superintendent at the above address.

Mrs. Charles must take the earliest opportunity of explaining her complaints against the Paddington Infirmary in the circular issued to voters. As a rule we are all for women guardians, but voters should be careful whom they elect.

The magistrate at the Southwark Police Court has imposed fines amounting to upwards of £21, on the owner of a house near the Blackfriars Road, for failing to comply with the order of the sanitary authority to put the premises in proper sanitary condition.

The Religious Tract Society has just contributed £20 towards the cost of "The Pilgrim's Progress" for the blind in the island of Formosa. It will be published in the Braille type, which the Chinese themselves are now able to prepare, and will be the eighty-fifth version of John Bunyan's book.

The Analyst of metropolitan water says the proportion of organic matter is—Kent, 0·5; New River, 1·3; Colne Valley, 1·4; Tottenham, 1·7; Grand Junction, 2·1; Southwark and Lambeth, 2·4; East London, 2·6; West Middlesex, 2·; and Chelsea, 3·5. The water was efficiently filtered.

The Women's Trades' Association is now established at an East-End centre—128, Mile End Road. Unions are already formed among the female workers of the following trades: Bookbinders, upholstresses, shirt-makers, tailoresses, match-makers, cigar-makers, confectioners, rope-makers, and mantle-workers.

In Queen's Bench, last week, in the case of Martin v. Stubbs, the parties being doctors at Hammersmith, a verdict of £200 damages was given in favour of the plaintiff as damages for libel contained in a medical certificate given by the defendant that a man had died from an overdose of turpentine given by "another doctor."

The Duke and Duchess of Westminster have issued invitations to a meeting at Grosvenor House on Friday afternoon next, when details will be given respecting the holiday homes and other beneficent work of the Ragged School Union. The Baroness Burdett-Coutts, Lord Kinnaird, Earl Compton, M.P., and Canon Fleming are expected to be present.

The Flower Girls' Guild will very shortly send forth into our West-End streets a band of picturesquely clad women whose attire will form a suitable set-off to their beautiful stock-in-trade, instead of the old familiar contrast of dirt and squalor. On May Day the work of the guild will be inaugurated by a tea given to the women, to which friends of the movement will be invited.

